# Evidenz‐ und konsensbasierte (S3) Leitlinie: Lichen sclerosus

**DOI:** 10.1111/ddg.70000x

**Published:** 2026-04-08

**Authors:** Gudula Kirtschig, Linn Woelber, Andreas Günthert, Karl Becker, Alexandra Ciresa‐König, Bettina Fischer, Susanne Fricke‐Otto, Claudia Günther, Christine Hirchenhain, Narayani Helga Köllmann, Alexander Kreuter, Bartosz Malisiewicz, Felix Neis, Dan mon O'Dey, Hagen Ott, Martin Promm, Regina Renner, Anne‐Rose Schardt, Ines Schweizer, Raimund Stein, Jan Ter‐Nedden, Gerda Trutnovsky, Agnes Wand, Gerhard Weyandt, Ricardo N. Werner, Maria Kinberger

**Affiliations:** ^1^ Medbase Health Centre Frauenfeld Frauenfeld Schweiz; ^2^ Dysplasiezentrum Hamburg am Krankenhaus Jerusalem Universitätsklinikum Hamburg‐Eppendorf Hamburg Deutschland; ^3^ gyn‐zentrum Luzern Cham Kriens Luzern Schweiz; ^4^ Kinderchirurgische Praxis Bonn Bonn Deutschland; ^5^ Universitätsklinik für Gynäkologie und Geburtshilfe Med. Universität Innsbruck Österreich; ^6^ Verein Lichen sclerosus Schweiz Dottikon Schweiz; ^7^ Pädiatrische Endokrinologie und Diabetologie Kindernotaufnahme Kinderschutzteam Helios‐Klinikum Krefeld Krefeld Deutschland; ^8^ Universitätsklinikum Carl Gustav Carus Dresden Klinik und Poliklinik für Dermatologie Dresden Deutschland; ^9^ Universitätsklinikum Carl Gustav Carus Dresden Klinik und Poliklinik für Frauenheilkunde und Geburtshilfe Dresden Deutschland; ^10^ Klinik für Dermatologie Venerologie und Allergologie Helios St. Elisabeth Klinik Oberhausen Oberhausen Deutschland; ^11^ Klinik für Dermatologie Venerologie und Allergologie Helios St. Johannes Klinik Duisburg Duisburg Deutschland; ^12^ Main Medic. All Praxis Main Deutschland; ^13^ Klinik für Gynäkologie und Geburtshilfe Universitätsklinikum Tübingen Tübingen Deutschland; ^14^ Luisenhospital Aachen/Klinik für Plastische Rekonstruktive und Ästhetische Chirurgie Handchirurgie Zentrum für Rekonstruktive Chirurgie weiblicher Geschlechtsmerkmale Aachen Deutschland; ^15^ MUC‐iSPZ Hauner LMU Zentrum für Entwicklung und komplex chronisch kranke Kinder Ludwig‐Maximilian‐Universität München München Deutschland; ^16^ Barmherzige Brüder Regensburg Klinik für Kinderurologie Regensburg Deutschland; ^17^ Hautarztpraxis Esslingen Esslingen Deutschland; ^18^ Gemeinschaftspraxis Ihre Frauenärztinnen im Taunusstein Taunusstein Deutschland; ^19^ Praxis für Sexual‐ und Psychotherapie Luzern Schweiz; ^20^ Zentrum für Kinder‐ Jugend‐ und rekonstruktive Urologie Universitätsklinikum Mannheim Mannheim Deutschland; ^21^ Dermatopathologisches und Pathologisches Einsendelabor Hamburg Hamburg Deutschland; ^22^ Meduni Graz Universitätsklinik Klinik Frauenheilkunde und Geburtshilfe Graz Österreich; ^23^ Alice‐Salomon‐Hochschule Berlin Berlin Deutschland; ^24^ Klinik und Poliklinik für Dermatologie Allergologie und Venerologie Klinikum Bayreuth Bayreuth Deutschland; ^25^ Division of Evidence‐Based Medicine (dEBM) Klinik für Dermatologie Venerologie und Allergologie Charité – Universitätsmedizin Berlin corporate member of Freie Universität Berlin and Humboldt‐Universität zu Berlin Berlin Deutschland

**Keywords:** Evidenzbasierte Leitlinie, Leitlinie, Lichen sclerosus, guideline, lichen sclerosus

## Abstract

Die deutschsprachige, konsens‐ und evidenzbasierte S3‐Leitlinie zum Lichen sclerosus (LS) wurde unter Federführung der *Deutschen Dermatologischen Gesellschaft e.V*. (DDG) und der *Deutschen Gesellschaft für Gynäkologie und Geburtshilfe e.V*. (DGGG) auf Basis der europäischen „EuroGuiDerm Guideline on lichen sclerosus“ entwickelt. Besonderes Augenmerk galt dabei der Anpassung an die medizinischen Versorgungssituation im deutschsprachigen Raum. Das interdisziplinäre Leitliniengremium umfasste 24 Experten aus 16 Fachgesellschaften und schloss auch Patientenvertreter aktiv in den Entwicklungsprozess ein.

Die Leitlinie enthält unter anderem umfassende Empfehlungen zur Diagnostik, zum Patientenmanagement, zur Nachsorge und Patientenschulung sowie zur Therapie des genitalen und extragenitalen LS bei Frauen, Männern, Mädchen und Jungen. Unabhängig von Alter und Geschlecht gelten ultrapotente oder potente topische Glukokortikoide in Kombination mit pflegenden Externa weiterhin als Standardtherapie des genitalen LS. Bei unzureichendem Ansprechen auf diese Therapie ist bei männlichen Patienten mit LS bedingter Phimose eine Zirkumzision mit vollständiger Entfernung der Vorhaut indiziert. Für den extragenitalen LS wird ergänzend zur topischen Therapie eine Phototherapie mit UV‐Licht empfohlen. Topische Calcineurininhibitioren können als Zweitlinientherapie zum Einsatz kommen.

## EINLEITUNG

Bei der vorliegenden deutschsprachigen Leitlinie zu Lichen sclerosus handelt es sich um eine adaptierte Fassung der „EuroGuiDerm Guideline on lichen sclerosus“ von Kirtschig G. et al.^1^, ^2^ Die übersetzte europäische Leitlinie wurde gezielt um zentrale Inhalte und Aspekte ergänzt, die für den deutschsprachigen Versorgungsraum besonders relevant sind.

Die vorliegende Publikation stellt eine Kurzfassung der Leitlinie dar. Einige Kapitel sind in dieser Kurzversion nicht enthalten. Der Hintergrundtext der enthaltenen Kapitel ist zum Teil stark gekürzt. Die vollständige Langfassung, der Leitlinienreport sowie der Evidenzreport sind auf der Webseite der AWMF einsehbar: https://register.awmf.org/de/leitlinien/detail/013‐105

## METHODIK

Für weitere Informationen siehe auch den Leitlinienreport oder die Langfassung der Leitlinie.

Zur standardisierten Darstellung der Empfehlungen wurden die in Tabelle 1 dargestellten Begrifflichkeiten und Symbole verwendet. Alle Empfehlungen wurden für die deutschsprachige AWMF‐S3‐Leitlinie neu formuliert, diskutiert und konsentiert.

**TABELLE 1:** Empfehlungsstärken – Wortwahl, Symbolik und Interpretation, modifiziert nach Kaminski‐Hartenthaler et al.^3^

Empfehlungsstärke	Wortwahl	Symbol	Interpretation
*Starke* Empfehlung für eine Vorgehensweise	„…soll…“	**↑↑**	Wir sind der Auffassung, dass alle oder fast alle informierten Menschen diese Entscheidung treffen würden. Kliniker müssen sich weniger Zeit für die Entscheidungsfindung gemeinsam mit dem Patienten nehmen.
*Schwache* Empfehlung für eine Vorgehensweise	„…sollte…“	**↑**	Wir sind der Auffassung, dass die meisten informierten Menschen, ein substanzieller Anteil jedoch nicht, diese Entscheidung treffen würden. Kliniker müssen mehr Zeit aufwenden, um sicherzustellen, dass die Wahl des Verfahrens die Werte und Präferenzen des individuellen Patienten widerspiegelt.
Offene Empfehlung	„… kann erwogen werden …“	⇔	Zurzeit kann eine Empfehlung für oder gegen eine Vorgehensweise aufgrund bestimmter Gegebenheiten nicht getroffen werden (zum Beispiel fehlende oder unzureichende Evidenz, unklares Nutzen‐Risiko‐Verhältnis).
*Schwache Empfehlung* gegen eine Vorgehensweise	„…sollte nicht…“	**↓**	Wir sind der Auffassung, dass die meisten informierten Menschen, ein substanzieller Anteil jedoch nicht, diese Entscheidung treffen würden.
*Starke* Empfehlung gegen eine Vorgehensweise	„…soll nicht…“	**↓↓**	Wir sind der Auffassung, dass alle oder fast alle informierten Menschen diese Entscheidung treffen würden.

## DEFINITION DER KRANKHEIT

Der Lichen sclerosus (LS) ist eine entzündliche Hauterkrankung, die typischerweise den anogenitalen Bereich betrifft, wo sie Juckreiz und Schmerzen verursacht. Die Erkrankung kann bei Frauen und Männern zu sexuellen Funktionsstörungen und Miktionsstörungen führen, sie kann jedoch auch asymptomatisch verlaufen. Zu den Frühsymptomen zählen unter anderem urologische Beschwerden, Dyspareunie sowie postkoitale Rhagaden.^4^, ^5^ Die ersten Anzeichen von LS sind in der Regel eine Aufhellung der genitalen Haut, manchmal Rötung und Ödeme; im Verlauf können Fissuren, Vernarbungen, Atrophie und Fusionen von Strukturen auftreten (Tabelle 2). Der LS bedingt im Erwachsenenalter ein erhöhtes Risiko für das Entstehen lokaler Karzinome, üblicherweise Plattenepithelkarzinome. Bei einer Minderheit der Patienten tritt die Krankheit auch außerhalb des Genitalbereichs auf. Der Verlauf des LS ist in der Regel chronisch. Die Behandlungsergebnisse bleiben oft, insbesondere bei verzögerter Einleitung einer adäquaten Therapie, unbefriedigend, da es trotz Behandlung zu einer beeinträchtigenden Sklerosierung der Haut kommen kann.^6^, ^7^, ^8^, ^9^ Es gibt Hinweise darauf, dass LS bei Männern nach einer Zirkumzision in Remission gehen kann, jedoch fehlen gute Studien dazu.

**TABELLE 2:** Symptome und klinische Zeichen des Lichen sclerosus.

− **Symptome** − Juckreiz (hauptsächlich bei genitalem LS bei Mädchen und Frauen) − Schmerzen / Brennen − Reizung − Gefühl der Trockenheit − Dysästhesie − Obstipation, bei perianaler Beteiligung, besonders bei Mädchen − Dyspareunie oder Apareunie (gestörte sexuelle Funktion) − Dysurie (Schmerzen, veränderter Harnstrahl) − Blasenschmerzen (abakterielle Zystitis) − LS kann asymptomatisch sein	− **Klinische Zeichen** − Ödem (Schwellung der Haut) − Leichtes Erythem (Rötung) − Hyperkeratose (weiße verdickte Haut; Hyperkeratose in der Histologie) − Sklerose (feste, gelblich weißliche Haut, zum Beispiel bei Phimose; Frenulum breve, dermale Sklerose in der Histologie) − Blässe (blassweiße Bereiche; das histologische Korrelat ist nicht beschrieben) − Atrophe Haut (faltige Haut; epidermale Atrophie in der Histologie) − Fissuren (Hautfragilität, Verlust der Elastizität, was zu Rissen in der Haut führt) − Erosionen − Blasenbildung ist sehr selten − Ekchymosen/Purpura sind bei genitalem LS häufig (aufgrund von fragilen, sklerotischen und erweiterten Blutgefäßen) − Veränderungen können auf die Vulva beschränkt sein oder den perianalen Bereich einbeziehen, wodurch eine „Acht‐förmige Verteilung“ entsteht − Narbenbildung kann zu architektonischen Veränderungen führen (zum Beispiel Resorption der Labia minora, Fusion in der Mittellinie mit verborgener, aber nicht Verlust der Klitoris bei Mädchen und Frauen und zum Beispiel Phimose, enge Harnröhrenöffnung und sklerotisches Frenulum breve bei Jungen und Männern) − Follikuläres Plugging (bei extragenitalem LS)

Synonyme wie Kraurosis vulvae, Balanitis xerotica obliterans und *white‐spot disease* gelten als veraltet und sollten nicht mehr verwendet werden. Das Suffix *et atrophicus* wurde fallengelassen, da einige Fälle von LS mit einem hypertrophen, anstatt atrophen Epithel assoziiert sind.

Der LS hat einen erheblichen Einfluss auf die Lebensqualität der betroffenen Patienten, daher ist es wichtig, das Bewusstsein für diese nicht selten auftretende Krankheit zu verbessern, um sie frühzeitig diagnostizieren und behandeln zu können.^10^, ^11^, ^12^

## KLINISCHES ERSCHEINUNGSBILD UND FOLGEN DER ERKRANKUNG

Der LS ist eine chronische Erkrankung mit verschiedenen und schwankenden Symptomen. Bei Mädchen und Frauen sind die anfänglichen Beschwerden bei genitalem LS vor allem Juckreiz oder Schmerzen, Männer klagen hingegen eher über sexuelle Dysfunktion.^4^

### Klinische Präsentation bei Mädchen und Frauen

**ABBILDUNG 1:**
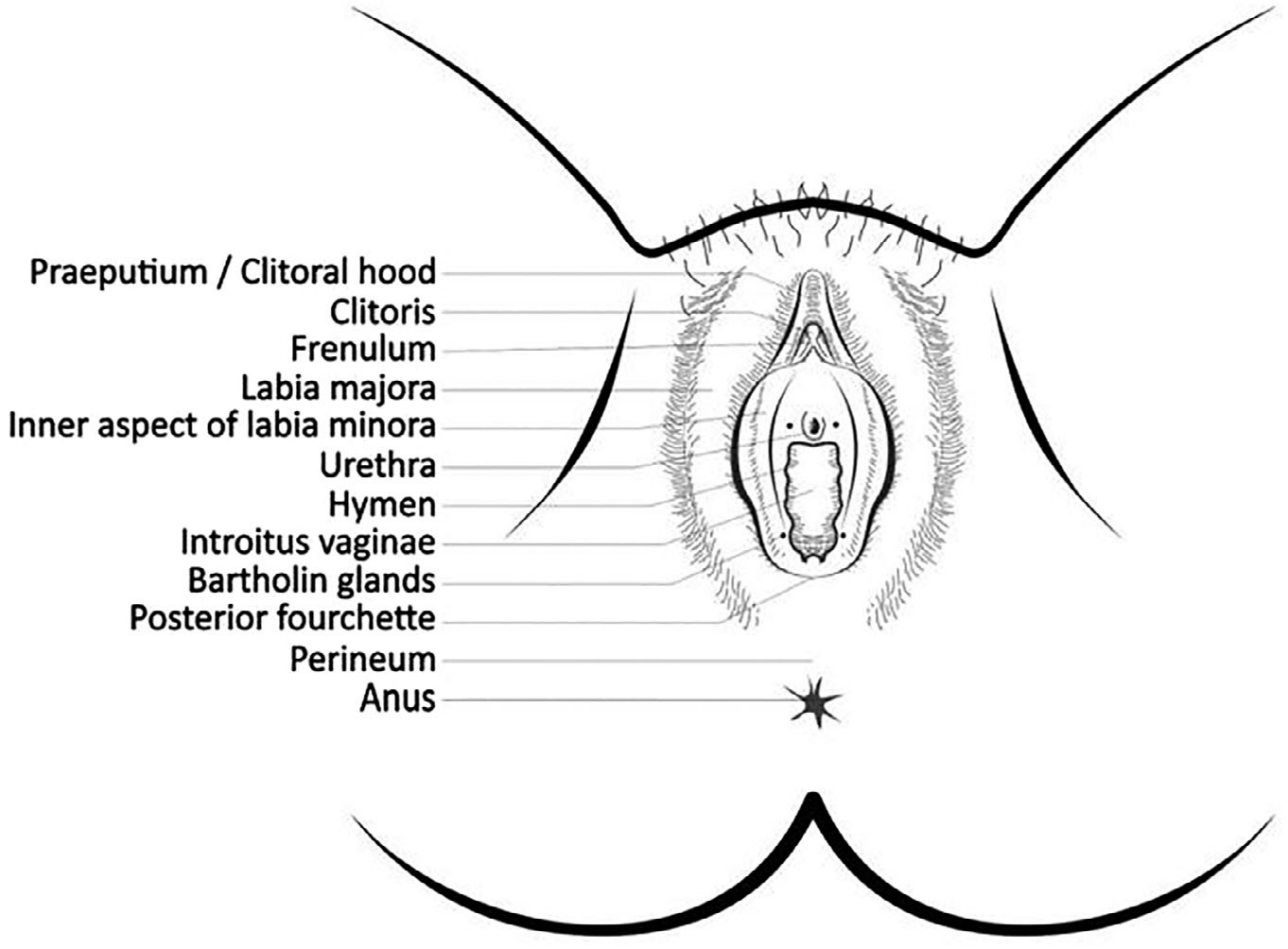
Äußere weibliche Genitalien, übernommen aus Gynäkologische Dermatologie.^1^ ^3^

**ABBILDUNG 2:**
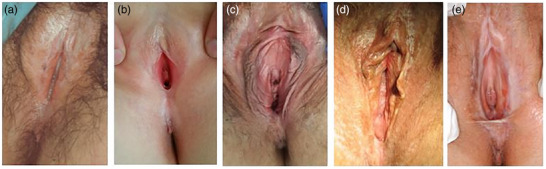
Typische Veränderungen des äußeren weiblichen Genitales bei Lichen sclerosus: (a) Lichen sclerosus bei zwei (prä‐)pubertären Mädchen mit ausgeprägter Hyperkeratose um die Klitoris und in den Interlabialfalten.^1^ ^4^ (b) Leichte Hyperkeratose um die Klitoris und an der hinteren Vaginalkommissur. (c) Früher Lichen sclerosus ohne sklerotische Veränderungen; die Patientin klagte über Juckreiz und Fissurbildung beim Geschlechtsverkehr, das linke Labium minus ist verkürzt. (d) Weiße Läsionen um die Klitoris sowie Hyperkeratose an der hinteren Vaginalkommissur. (e) Fusion der Labia minora oberhalb der Klitoris mit verborgener Klitoris und atrophen Labia minora als Ausdruck architektonischer Veränderungen; Ekchymose in der linken Interlabialfalte.

Charakteristischerweise sind bei Frauen die Interlabialfalten, die Labia minora, die Glans clitoridis, das Präputium und die Präputialöffnung, die Commissura posterior und das Perineum (oft bei Mädchen) betroffen. Die Labia majora und die Harnröhrenöffnung können ebenfalls involviert sein (Abbildungen 1, 2).

### Klinische Präsentation bei Jungen und Männern

**ABBILDUNG 3:**
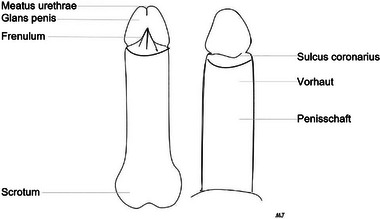
Penile Strukturen.

**ABBILDUNG 4:**
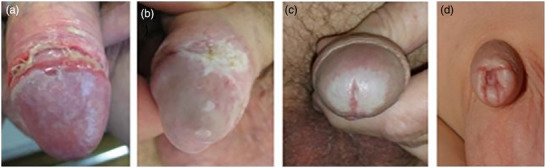
Lichen sclerosus bei Jungen und Männern. (a) Erosiver Lichen sclerosus am Sulcus coronarius und weiße Läsionen an der Glans penis. (b) Hyperkeratotische Plaque am Sulcus coronarius. (c) Blässe beziehungsweise Sklerose der Glans penis mit vom Lichen sclerosus betroffener Harnröhrenöffnung. (d) Sekundäre Phimose bei einem Jungen.

Bei Jungen und Männern tritt der LS in der Regel auf der Glans penis, dem Sulcus coronarius, am Meatus urethrae und/oder der Vorhaut auf, bevorzugt perifrenulär (Abbildungen 3, 4). Dies kann zu einer Phimose in einer zuvor zurückziehbaren Vorhaut oder zu Verwachsungen der Vorhaut mit der Glans penis führen, was Dysurie oder Schmerzen bei der Erektion verursachen kann. Eine sekundäre Phimose weist, insbesondere bei Kindern, auf LS als Ursache hin. Selten sind der Penisschaft, die perineale, skrotale oder perianale Haut betroffen. Eine Meatusstenose kann zu Miktionsproblemen führen. Wenn der LS die Harnröhre betrifft, stellt dies eine schwerwiegende Komplikation dar. Man geht davon aus, dass LS bei nicht oder spät zirkumzidierten Männern häufiger auftritt als bei Männern, die bereits kurz nach der Geburt beschnitten wurden.^15^, ^16^

### Klinische Präsentation des extragenitalen LS

**ABBILDUNG 5:**
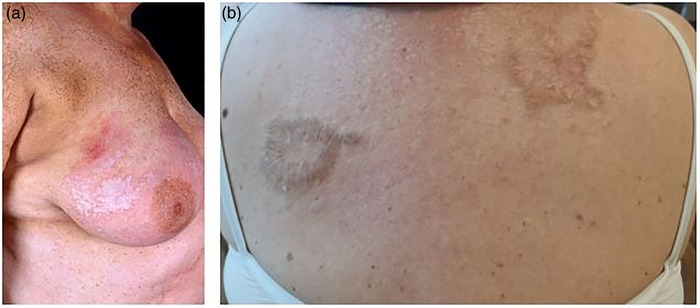
Extragenitaler Lichen sclerosus. (a) Sklerotische Läsion an der Mamma. (b) Sklerotische und hyperkeratotische Läsion am Rücken.

LS, der ausschließlich extragenitale Haut befällt, ist selten und wurde bei etwa 6 % aller betroffenen Frauen berichtet (Abbildung 5).^17^ Beteiligung der Kopfhaut, einschließlich bullöser Varianten und vernarbender Alopezie, ist selten.^18^, ^19^, ^20^ Es wurde darüber berichtet, dass LS in extrem seltenen Fällen auch an der Mundschleimhaut und den Nägeln auftreten kann.^21^, ^22^, ^23^, ^24^, ^25^, ^26^

Im Gegensatz zu der üblicherweise genitalen Manifestation des LS in der westlichen Hemisphäre berichtet eine irakische Gruppe über circa 10 % genitale Manifestation und circa 90 % extragenitale Läsionen, die hauptsächlich an den Extremitäten und dem Rumpf bestehen, aber auch die Lippen und den Skalp betreffen.^27^

### Symptome und klinische Zeichen des LS

Ärzte sollten mit den verschiedenen klinischen Zeichen des LS vertraut sein. Einige der klinischen Zeichen repräsentieren frühe, reversible Anzeichen, andere sind permanente, nicht reversible Zeichen des LS.

*Reversible Merkmale*:

− Fissuren/Erosionen sind Längsrisse/oberflächliche Wunden der Hautoberfläche.
− Ekchymosen sind Blutungen in der Haut.
− Hyperkeratosen sind Plaques aus hellweißer Haut mit einem „pudrigen“ Aussehen.

*Irreversible Merkmale*:

− Sklerose sind Bereiche aus gelblich/elfenbeinfarbener Haut mit einer glatten/wachsartigen/festen Textur.
− Blässe beziehungsweise blassweiße Haut unterscheidet sich von Hyperkeratosen dadurch, dass sie nicht „pudrig“ ist.

*Komplikationen*:

− Verlust des Selbstwertgefühls (zum Beispiel Sorge um das genitale Erscheinungsbild),
− Beeinträchtigung der Lebensqualität,
− Ängste und psychische Erkrankungen,
− sozialer Verlust (zum Beispiel Partner),
− Entwicklung von anogenitalen Karzinomen (tatsächliches Risiko <5 %),
− Entwicklung einer Pseudozyste an der Klitoris,
− sexuelle Dysfunktion,
− Miktionsstörung,
− genitale Dysästhesie.

## DIAGNOSESTELLUNG

Die Diagnose von LS basiert in der Regel auf dem charakteristischen klinischen Erscheinungsbild der Erkrankung. Klinische Scores sind in Entwicklung, aber noch nicht in der Praxis etabliert.^4^, ^28^, ^29^, ^30^, ^31^, ^32^, ^33^, ^34^

In typischen Fällen, wenn zum Beispiel Hyperkeratose oder Sklerose und Ekchymosen gesehen werden, ist keine Biopsie erforderlich. Die Durchführung einer Biopsie bei der ersten Vorstellung hilft jedoch, wenn die klinische Diagnose nicht eindeutig ist. Bei Kindern wird dies, aufgrund der damit verbundenen Belastung für das Kind, meist vermieden. Dennoch sollte, auch bei Kindern, eine Biopsie in Betracht gezogen werden, wenn die klinische Diagnose unsicher ist, eine Dysplasie oder ein Malignom vermutet wird oder wenn die Erstlinientherapie versagt, wobei hier zunächst die Therapieadhärenz überprüft werden muss. Bei Jungen findet üblicherweise bei einer sekundären Phimose eine Beschneidung statt im Rahmen derer eine feingewebliche Untersuchung der Vorhaut erfolgt, somit ist eine Biopsie hinfällig.

Wird eine Biopsie durchgeführt, ist die klinische und pathologische Korrelation entscheidend, dies gilt insbesondere für die frühen Phasen, in denen auch die Histologie unspezifisch sein kann.

### Histopathologie

Sollte eine Biopsie zur Diagnosestellung notwendig sein, sollte diese von einer unbehandelten und typischen Läsion mit weißlichem Aussehen (Hyperkeratose, „Blässe“ oder Sklerose) entnommen werden. Wenn sich diese nicht zeigen, kann zum Beispiel, wenn Fissuren oder Erosionen vorliegen, eine Biopsie am Ende einer Fissur entnommen werden, die typischerweise im Sulcus interlabialis auftreten oder auch im Randbereich einer Erosion (nicht aus der Mitte von erosiven Läsionen). Sind die Labia minora verkürzt, kann dies auf eine Krankheitsaktivität hinweisen und eine Biopsie an deren kaudalen Ende erfolgen.

Wenn vor der initialen Behandlung keine Biopsie durchgeführt wurde, wird eine dreiwöchige Behandlungspause (Pflegesubstanzen sind gestattet) für eine zuverlässige histologische Diagnose empfohlen. Wenn diese Therapiepause von Patienten nicht toleriert wird, ist es wichtig die Pathologen über die Art der Vorbehandlung zu informieren, da histologische Merkmale sich abhängig von der Dauer und Art der Behandlung verändern können.

Es ist jedoch zu beachten, dass histologische und klinische Merkmale des LS ein Spektrum umfassen. Dies kann zu falsch negativen histologischen Ergebnissen führen. In der Frühphase der Erkrankung können Merkmale, die bei anhaltender Krankheit gesehen werden, fehlen; so kann zum Beispiel die Hyalinisierung der oberen Dermis fehlen, was dazu führen kann, dass die Diagnose (auch) histologisch nicht sicher gestellt werden kann. In einigen Fällen können hypertrophe Formen des genitalen LS und Lichen planus ähnliche klinische und histologische Merkmale aufweisen, was die Unterscheidung schwierig macht. Wichtig ist es, präkanzeröse oder kanzerogene Läsionen auszuschließen. Plattenepithelkarzinome (PEK) bei LS entwickeln sich unabhängig von einer Infektion mit humanem Papillomavirus (HPV). Sie können innerhalb von Monaten entstehen.^35^ Daher sind neue hyperkeratotische Läsionen oder neu auftretende Erosionen und Ulzerationen verdächtig auf HPV‐unabhängige intraepitheliale Neoplasien der Vulva (und des Penis), die auch als differenzierte intraepitheliale Neoplasien der Vulva bezeichnet werden. Diese sollten immer biopsiert werden.

Zusammenfassend sollte eine größere Aufmerksamkeit für das klinische und histologische Spektrum des LS eine frühzeitigere Diagnose und Behandlung ermöglichen. Eine Biopsie, von erfahrenen (Dermato‐)Histopathologen interpretiert, ist hilfreich, um klinische Differenzialdiagnosen wie Lichen planus und Ekzeme (atopisch oder seborrhoisch), insbesondere bei Verdacht auf einen frühen LS, auszuschließen, sowie präkanzeröse und kanzeröse Läsionen zu erkennen.

### Diagnostik bei Kindern

Besondere Aufmerksamkeit ist bei Kindern mit anogenitalen Hautveränderungen erforderlich, wobei zu beachten ist, dass Kinder keine *kleinen Erwachsenen* sind.

Bei der Untersuchung und Behandlung von Kindern sollte eine kinderfreundliche Umgebung hergestellt werden. Die Untersuchung sollte von erfahrenen Ärzten durchgeführt werden, die mit der Erkrankung, der Anatomie der Genitalien bei Kindern und mit der Kommunikation mit Kindern vertraut sind.

Mädchen sollten vorzugsweise auf einer Patientenliege und nicht auf einem gynäkologischen Stuhl untersucht werden, um eine kindgerechte Untersuchungsatmosphäre zu gewährleisten.

## EINFÜHRUNG IN DAS THERAPEUTISCHE MANAGEMENT

### Ziele der Behandlung

Neben individuellen Behandlungszielen sollte der therapeutische Ansatz im Allgemeinen multidimensional sein und folgende Ziele verfolgen:

1. Schnelle Verbesserung von Symptomen wie Juckreiz, Schmerzen oder Brennen.
2. Erhaltung oder Verbesserung der Lebensqualität, einschließlich des Sexuallebens und der problemlosen Miktion.
3. Kontrolle von klinischen Krankheitszeichen, um beispielsweise Narbenbildung, Gewebe‐Resorption, Hautatrophie und maligne Transformation zu vermeiden.
4. Reduzierung von Krankheitsschüben.
5. Heilung von LS bei Jungen und Männern.

Für alle Patienten müssen die Behandlungsziele individuell bewertet werden. Im Verlauf der Krankheit müssen diese Ziele von Zeit zu Zeit neu bewertet werden.

## HAUTPFLEGE UND ALLGEMEINE THERAPIEEMPFEHLUNGEN

**Table:** 

Bei Patienten mit LS *sollen* Salben anstelle von Cremes oder Gelen verwendet werden.	**↑↑**	100 % Konsens konsensbasiert

**Table:** 

Triggerfaktoren (mechanische Reizungen wie Traumata, unnötige chirurgische Eingriffe, Piercings) und Irritation (wie übermäßige Wasserexposition oder exzessive Verwendung von Reinigungsprodukten, synthetische und enge Kleidung, Verwendung von Feuchttüchern) *sollten* an betroffenen LS‐Lokalisationen vermieden werden.	**↑**	100 % Konsens konsensbasiert

**Table:** 

Bei Patienten mit LS *sollten* Inkontinenzeinlagen und Unterwäsche regelmäßig gewechselt werden und Urininkontinenz so gut wie möglich behandelt werden.	**↑**	100 % Konsens konsensbasiert

**Table:** 

Die Verwendung von pflanzlich basierten Topika, topischen Antihistaminika, topischen Anästhetika und parfümierten Topika *sollte* bei Patienten mit LS *vermieden* werden, da ein erhöhtes Risiko einer Kontaktsensibilisierung besteht.	**↓**	> 75 % Konsens konsensbasiert

**Table:** 

Bei klinischer Verschlechterung des LS oder bei Zunahme der Symptome wie Juckreiz oder Schmerzen *sollen* eine adäquate Therapie sichergestellt und andere Ursachen wie Kontaktallergien, Infektionen und maligne Transformation berücksichtigt beziehungsweise ausgeschlossen werden.	**↑↑**	100 % Konsens konsensbasiert

## TOPISCHE THERAPIE MIT EMOLLIENZIEN

**Table:** 

Zusätzlich zur Standardtherapie *soll* bei *Frauen und Mädchen* mit genitalem LS eine Basistherapie mit Emollienzien erfolgen.	**↑↑**	100 % Konsens Konsensbasiert*
Zusätzlich zur Standardtherapie *sollte* bei *Männern und Jungen* mit genitalem LS eine Basistherapie mit Emollienzien erfolgen.	**↑**	
Zusätzlich zur Standardtherapie *sollte* bei *extragenitalem LS* eine Basistherapie mit Emollienzien erfolgen.	**↑**	
*Aufgrund der geringen Evidenzlage entschied sich das Leitliniengremium hier rein Konsens‐basierte Empfehlungen trotz systematischer Evidenzaufbereitung (siehe Evidenzbericht) auszusprechen.		

### Wirksamkeit und Wirkmechanismus

Unter Emollienzien versteht man Pharmazeutika, die die Haut weich und geschmeidig machen. Sie wirken durch „Auffüllen“ von Spalten zwischen Korneozyten. Durch dieses „Abdichten“ der Haut kommt es zu einem geringeren transdermalen Feuchtigkeitsverlust. Subjektiv verspüren Menschen durch das Auftragen von Emollienzien weniger Trockenheit und glattere weichere Haut. Die Wirkung ist insbesondere bei Menschen mit bestimmten Dermatosen wie atopischer Dermatitis und Psoriasis vulgaris nachgewiesen.

Beispiele für Emollienzien sind: Lanolin, Fettsäuren und deren Derivate, speziell Paraffine unter anderem verarbeitet zu Vaseline, pflanzliche Öle aber auch Propylenglycolderivate.

Emollienzien können nach einer initialen Behandlung mit topischen Glukokortikoiden bei LS eine zusätzliche Linderung der Symptome bewirken.

Eine Studie mit initialer Anwendung eines topischen Glukokortikoids und anschließender Erhaltungsbehandlung mit täglicher Anwendung einer *Cold Cream* (Unguentum leniens) zeigte bei Frauen mit LS, dass die initiale Symptomlinderung auch während der Erhaltungstherapie aufrechterhalten werden konnte. Allerdings kann daraus nicht eindeutig abgeleitet werden, ob dies die Wirkung der Emollienzien oder eine Langzeitwirkung des Glukokortikoides war.^36^

Eine randomisierte Studie mit topischer Vitamin‐E‐Creme im Vergleich zu einem Emolliens nach einer Initialbehandlung mit einem topischen Glukokortikoid zeigte keine Unterschiede in den Rückfallraten über einen Zeitraum von einem Jahr, daher scheint Vitamin E keinen zusätzlichen Vorteil gegenüber Emollienzien zu haben.^37^

### Dosierung: Akut und Erhaltung

Bezüglich der optimalen Dosierung gibt es bislang keine Daten. Aufgrund unserer Erfahrung empfehlen wir mindestens zweimal täglich Emollienzien aufzutragen, um die Hautbarriere zu stärken und sie gegen irritierende äußere Faktoren wie Urin, Seifen, vaginalem Ausfluss, Schweiß, Gleitmittel, Sperma und Reibung beim Geschlechtsverkehr zu schützen. Wenn die Labien bei der Miktion brennen, kann das Auftragen von Emollienzien vor der Miktion helfen, den Hautkontakt mit dem Urin zu verringern. Um eine verdünnende Wirkung der topischen Glukokortikoide zu vermeiden, sollten die Emollienzien zeitlich versetzt zu den Glukokortikoiden aufgetragen werden.

### Sicherheit und besondere Hinweise

Im Allgemeinen sind Emollienzien sehr gut verträglich. Es wurden bislang keine Sicherheitsbedenken geäußert. In seltenen Fällen können sie jedoch zu reizender oder allergischer Kontaktdermatitis führen. So sind beispielsweise Zusatzstoffe wie Benzoesäure, Benzalkoniumchlorid, Polyethylenglykol oder Natriumlaurylsulfat (SLS) bekannte Irritanzien,^38^, ^39^ die Bestandteil von Emollienzien sein können. Ebenso kann Benzoesäure in Emollienzien als Allergen wirken. Dies gilt auch für Lanolin, Jojobaöl, Propylenglykol‐Duftstoffe und Perubalsam, die zu den häufigsten Allergenen gehören.^38^, ^40^ Daher sollten nur duftstofffreie Emollienzien verwendet werden.

## TOPISCHE UND INTRALÄSIONALE THERAPIE MIT GLUKOKORTIKOIDEN

**Table:** 

Bei *Frauen* mit genitalem LS *soll* eine topische Therapie mit Glukokortikoiden der Klasse III oder IV erfolgen.	**↑↑**	100 % Konsens evidenz‐ und konsensbasiert, siehe Evidenzbericht
Bei *Mädchen* mit genitalem LS *soll* eine topische Therapie mit Glukokortikoiden der Klasse III* oder IV* erfolgen.	**↑↑**	
Bei *Männern* mit genitalem LS *soll* eine topische Therapie mit Glukokortikoiden der Klasse III oder IV erfolgen.	**↑↑**	
Bei *Jungen* mit genitalem LS *soll* eine topische Therapie mit Glukokortikoiden der Klasse III* oder IV* erfolgen.	**↑↑**	
Bei Patienten mit *extragenitalem LS sollte* eine topische Therapie mit Glukokortikoiden der Klasse III* oder IV* erfolgen.	**↑**	
*Nicht alle Präparate sind für Kinder jeder Altersklasse zugelassen.		

**Table:** 

Bei Patienten mit LS *sollen* Glukokortikoide in Salben‐Grundlage anstelle von Cremes‐ oder Lotions‐Grundlage verwendet werden.	**↑↑**	100 % Konsens konsensbasiert

**Table:** 

Bei *Frauen* mit hyperkeratotischem genitalem LS *kann* eine Therapie mit intraläsionalen Glukokortikoiden *erwogen werden*, wenn die LS‐Läsionen nicht auf eine topische Therapie angesprochen haben (vorausgesetzt, dass eine Neoplasie ausgeschlossen wurde).	⇔	100 % Konsens evidenz‐ und konsensbasiert, siehe Evidenzbericht
Bei *Mädchen soll* eine Therapie mit intraläsionalen Glukokortikoiden zur Behandlung des genitalen LS *nicht* erfolgen.	**↓↓**	
Bei *Männern* mit hyperkeratotischem genitalem LS *kann* eine Therapie mit intraläsionalen Glukokortikoiden *erwogen werden*, wenn die LS Läsionen nicht auf eine topische Therapie angesprochen haben (vorausgesetzt, dass eine Neoplasie ausgeschlossen wurde).	⇔	
Bei *Jungen* mit genitalem LS *kann* im Rahmen einer vorhauterhaltenden Operation eine Therapie mit intraläsionalen Glukokortikoiden in Narkose *erwogen werden*.	⇔	

### Topische Glukokortikoide der Klasse III und IV

#### Wirksamkeit und Wirkmechanismus

Aufgrund ihrer nachgewiesenen Wirksamkeit und Sicherheit werden topische Glukokortikoide der Klasse III oder IV wie Clobetasolpropionat 0,05 % oder Mometasonfuroat 0,1 % Salbe (oder Creme) als bevorzugte Behandlung empfohlen, sowohl bei akuten Schüben als auch in der Erhaltungstherapie.^41^, ^42^, ^43^

Topische Glukokortikoide wirken als entzündungshemmende und antifibrotische Therapeutika. Sie beeinflussen mehrere verschiedene Signalwege, indem sie aktivierte (pro)‐inflammatorische Gene hemmen.^44^, ^45^, ^46^ Zudem haben sie auch eine schnelle juckreizstillende Wirkung.

Topische Glukokortikoide der Klasse III oder IV bewirken eine rasche Verbesserung der klinischen Zeichen und der subjektiven Symptome (in der Regel in weniger als 10 Tagen).

Cremes und Salben sind die am häufigsten verwendeten Grundlagen, da sie sich leicht verteilen lassen und gut auf der Schleimhaut haften. Unserer Erfahrung nach ist es jedoch sinnvoll Patienten mehrere Grundlagen ausprobieren zu lassen, um individuell die wirksamste und angenehmste Behandlungsform zu finden; dies fördert die Adhärenz. Es gilt jedoch zu erwähnen, dass Salben in der Regel weniger brennen, weniger Kontaktallergene beinhalten, einen besseren Barriereschutz bieten und eine bessere Penetration des Wirkstoffes ermöglichen.

Die Wirksamkeit topischer Glukokortikoide der Klasse III und IV scheint bei der Behandlung von genitalem LS vergleichbar zu sein. Die meisten Studien wurden mit Glukokortikoiden der Klasse IV (Clobetasolpropionat 0,05 %) durchgeführt. In bestimmten Situationen können jedoch moderne Glukokortikoide der Klasse III wie Mometasonfuroat bevorzugt werden. Dies ist bei Kindern der Fall, da bei diesen die Haut dünner ist und dementsprechend steroidbedingte Nebenwirkungen bei Klasse‐IV‐Präparaten häufiger auftreten können. Auch in der Schwangerschaft, bei der eine Resorption des Glukokortikoids aus Sicherheitsgründen (Risiko der Wachstumsretardierung) zu vermeiden ist, können Klasse III Glukokortikoide bevorzugt werden.

#### Mädchen und Frauen mit genitalem LS

Etwa 60 % bis 70 % der Patientinnen mit LS erreichen eine vollständige Remission ihrer Symptome nach einer dreimonatigen Behandlung mit Clobetasolpropionat 0,05 %, welches in der Regel einmal täglich angewendet wird.^8^, ^47^, ^48^ Ähnlich wirksam zeigte sich Mometasonfuroat 0,1 % einmal täglich nach 12 Wochen in einer direkten Vergleichsstudie. 59 % und 37 % der Patientinnen in der Clobetasolpropionat‐Gruppe und 67 % und 48 % in der Mometasonfuroat‐Gruppe erreichten eine Verbesserung der subjektiven beziehungsweise objektiven Bewertungsskalen um mindestens 75 %.^47^, ^48^

Es gibt Hinweise darauf, dass weniger potente Glukokortikoide (zum Beispiel Triamcinolon und Prednicarbat) ebenfalls als Erhaltungstherapie oder zur Behandlung mittelschwerer Schübe oder Rezidive wirksam sind.^45^, ^49^, ^50^

Es gibt keine vergleichenden randomisierten Studien bei Mädchen mit LS, jedoch zeigen nicht vergleichende Studien, dass die Behandlung mit Glukokortikoiden der Klasse III und IV bei der Unterdrückung von klinischen Zeichen und subjektiven Symptomen von LS wirksam sind.^51^, ^52^ Bei einigen Patientinnen geht der LS nach der Kindheit in Remission, der Verlauf ist jedoch variabel, weshalb eine engmaschige Nachsorge während und nach der Pubertät erforderlich ist, um ein Wiederauftreten des LS frühzeitig zu erkennen.^53^

#### Jungen und Männer mit genitalem LS

Sowohl Mometasonfuroat 0,1 % als auch Clobetasolpropionat 0,05 % sind wirksam bei der Behandlung von frühen und mittleren Erkrankungsstadien des penilen LS, jedoch ist die Heilungsrate unbekannt. In einer placebokontrollierten randomisierten Studie wurde die Wirksamkeit von Mometasonfuroat 0,05 % Salbe bei 40 Jungen mit penilem LS nach fünf Wochen Anwendung untersucht. Mometasonfuroat 0,1% verbesserte den klinischen Grad der Phimose bei 7 von 17 Jungen. Bei fortgeschrittener Erkrankung wurde keine Verbesserung festgestellt.^54^

Bei penilem LS besteht das Risiko der Entwicklung von Peniskarzinomen.^55^, ^56^, ^57^

#### Dosierung: Akut‐ und Erhaltungsbehandlung

In klinischen Studien wurde Clobetasolpropionat 0,05 % Creme oder Salbe 3 Monate lang ein‐ oder zweimal täglich aufgetragen oder schrittweise reduziert. Beispielsweise erfolgte einen Monat lang eine ein‐ oder zweimal tägliche Anwendung, dann 2 Monate lang eine einmal tägliche oder umtägige Anwendung. Manchmal in Abhängigkeit von der Schwere der klinischen Zeichen und der subjektiven Symptome oder dem Alter der Patienten.^41^, ^48^, ^58^

Einen Konsens über ein allgemeingültiges Standarddosierungsschema für die Behandlung von LS gibt es nicht (vergleiche obige Schemata).

Nur in seltenen Fällen kommt es zu einer vollständigen Remission ohne Rezidiv.

Es gibt Hinweise darauf, dass auch weniger potente Glukokortikoide (zum Beispiel Triamcinolon und Prednicarbat) als Erhaltungstherapie und zur Behandlung mittelschwerer Schübe oder Rezidive wirksam sind.^45^, ^49^, ^50^

#### Sicherheit

Nicht alle Präparate sind für Kinder aller Altersklassen zugelassen. Ein möglicher Off‐Label‐Gebrauch von topischen Glukokortikoiden sollte mit den Patienten und den Eltern besprochen werden.

Unerwünschte Wirkungen von topischen Glukokortikoiden werden selten beobachtet. Selten kommt es zu lokalen Reizungen und Brennen, insbesondere bei den ersten Anwendungen und wenn die Haut stark entzündet ist. Dies wird häufiger beobachtet, wenn Cremes statt Salben verwendet werden. Langfristig kann es zu Trockenheit, Hypopigmentierung und Hautatrophie kommen, insbesondere bei keratinisierter Haut. Allerdings können topische Glukokortikoide bei LS über Jahre hinweg ohne nennenswerte klinisch relevante unerwünschte Wirkungen angewendet werden (oft reichen 30 g bis 100 g Clobetasolpropionat 0,05 % für das gesamte Jahr in der Erhaltungsphase; in Akutphasen sollte eine Menge von 30 g pro Monat nicht überschritten werden). Die unerwünschten Wirkungen wie Stechen, Brennen und Xerosis werden meist eher mit dem Vehikel des topischen Glukokortikoides in Verbindung gebracht als mit dem Glukokortikoid selbst.^59^

Es ist wichtig, darauf hinzuweisen, wo die Topika aufgetragen werden müssen und wie viel Topikum zu verwenden ist. Zum Beispiel reicht eine Fingertip‐Einheit zur Behandlung der gesamten Vulva aus. Es ist nicht untersucht, ob die topischen Glukokortikoide nur auf die sichtbar von LS betroffenen Bereiche aufzutragen sind, zum Beispiel isoliert auf die Klitoris, die Labia minora, die interlabialen Sulci oder das Perineum oder auf den gesamten anogenitalen Bereich, der von LS betroffen sein kann. Sicherlich nicht sollten zum Beispiel die behaarte Labia majora behandelt werden, die so gut wie nie von LS betroffen ist. Topische Glukokortikoide dürfen nicht auf gesunde Haut aufgetragen werden, da sie dort bei kontinuierlicher Anwendung unerwünschte Wirkungen wie Erythem, Reizung und Hautatrophie verursachen.

### Intraläsionale Glukokortikoide

Die intraläsionale Injektion von Triamcinolonacetonid oder Dexamethason kann für einige Patienten mit LS eine Alternative zur Behandlung mit topischen Glukokortikoiden der Klasse IV sein.^60^, ^61^, ^62^

Intraläsionale Glukokortikoidinjektionen können bei mangelndem Ansprechen auf topische Glukokortikoide der Klasse III oder IV versucht werden, wenn zum Beispiel eine schlechte Penetration (wie bei stark hyperkeratotischen Läsionen) oder eine mangelnde Compliance vermutet wird.^61^ Bei atropher Haut oder bei kleinen Läsionen sind intraläsionale Glukokortikoidinjektionen zu vermieden, da das Gewebe geschädigt werden und ulzerieren kann. Die Therapie solle von einem Arzt mit Erfahrung in der intraläsionalen Glukokortikoidinjektion durchgeführt und überwacht werden.

## TOPISCHE THERAPIE MIT CALCINEURININHIBITOREN

**Table:** 

Bei *Frauen* mit genitalem LS *sollte* eine Therapie mit topischen Calcineurininhibitoren (off‐label) als zweite Wahl oder als zusätzliche topische Therapie erfolgen, wenn die topische Therapie mit Glukokortikoiden kontraindiziert oder nicht ausreichend wirksam ist.	**↑**	100 % Konsens evidenz‐ und konsensbasiert, siehe Evidenzbericht
Bei *Mädchen* mit genitalem LS *sollte* eine Therapie mit topischen Calcineurininhibitoren (off‐label) als zweite Wahl oder als zusätzliche topische Therapie erfolgen, wenn die topische Therapie mit Glukokortikoiden kontraindiziert oder nicht ausreichend wirksam ist.	**↑**	
Bei *Männern* mit genitalem LS *sollte* eine Therapie mit topischen Calcineurininhibitoren (off‐label) als zweite Wahl oder als zusätzliche topische Therapie erfolgen, wenn die topische Therapie mit Glukokortikoiden kontraindiziert oder nicht ausreichend wirksam ist.	**↑**	
Bei *Jungen* mit genitalem LS *sollte* eine Therapie mit topischen Calcineurininhibitoren (off‐label) als zweite Wahl oder als zusätzliche topische Therapie erfolgen, wenn die topische Therapie mit Glukokortikoiden kontraindiziert oder nicht ausreichend wirksam ist.	**↑**	
Bei Patienten mit *extragenitalem LS kann* eine Therapie mit topischen Calcineurininhibitoren (off‐label) *erwogen werden*.	⇔	

### Allgemeine Einführung

Zwei topische Calcineurininhibitoren (TCI), Pimecrolimus‐1 %‐Creme und Tacrolimus‐0,1 %‐ und ‐0,03 %‐Salbe, können *off‐label* zur Behandlung des LS verwendet werden.^63^, ^64^, ^65^, ^66^, ^67^, ^68^, ^69^, ^70^, ^71^, ^72^, ^73^, ^74^, ^75^, ^76^, ^77^ Zugelassen sind diese Präparate bislang nur für die atopische Dermatitis. Einige wenige randomisierte Studien vergleichen die Wirkung von TCIs mit Clobetasolpropionat 0,05 % bei LS der Vulva.^69^, ^78^, ^79^

### Wirksamkeit und Wirkmechanismus

Tacrolimus ist ein lipophiler immunsuppressiver Wirkstoff, der den *second messenger* Calcineurin hemmt und die Transkription von proinflammatorischen Zytokinen wie Interleukin (IL)‐2 und Interferon‐gamma blockiert. Durch die Hemmung von Calcineurin reduziert Tacrolimus zudem die Antigenpräsentation und die Aktivierung von T‐Zellen. Darüber hinaus beeinflusst es andere Zelltypen, die an Juckreiz und Entzündungsreaktionen beteiligt sind, wie etwa Mastzellen, eosinophile und basophile Granulozyten durch die Hemmung von IL‐3, IL‐8, IL‐13 und dem Granulozyten/Makrophagen‐Kolonie‐stimulierendem Faktor (GM‐CSF). Die Expression des FcεR1‐Rezeptors auf epidermalen antigenpräsentierenden Zellen wird reduziert. Während viele pharmakologische Effekte von topischem Calcineurininhibitoren denen von Glukokortikoiden ähneln, treten bei topischem Tacrolimus und Pimecrolimus keine Nebenwirkungen wie Hautatrophie und Teleangiektasien auf.^67^, ^68^

### Frauen mit genitalem LS

In einer doppelblinden, randomisierten Studie mit 38 Frauen mit histologisch gesichertem LS der Vulva erhielten die Patientinnen 12 Wochen lang eine Behandlung mit topischem Pimecrolimus 1 % oder Clobetasolpropionat 0,05 %. In beiden Gruppen zeigte sich eine ähnliche Verbesserung des Juckreizes und der Schmerzen.^78^ In der Reduktion des entzündlichen Prozesses war Clobetasolpropionat 0,05 % überlegen (p = 0,015).

Die Linderung von LS‐Symptomen durch Pimecrolimus wird auch in mehreren Fallserien bestätigt.^64^, ^80^, ^81^

In einer multizentrischen Phase‐II‐Studie wurde die Sicherheit und Wirksamkeit von Tacrolimus‐Salbe 0,1 % zur Behandlung von LS untersucht.^68^ Vierundachtzig Patienten (49 Frauen, 32 Männer und 3 Mädchen) zwischen 5 und 85 Jahren mit langjährigem, aktiven LS (79 mit anogenitalem und 5 mit extragenitalem LS) wurden zweimal täglich für 16 Wochen behandelt. Vierzehn Patienten brachen die Studie vorzeitig ab. Eine Remission des aktiven LS wurde bei 43 % (ITT 36 %) der Patienten nach 24 Wochen Behandlung erreicht. Eine Teilremission wurde bei 34 % (ITT 29 %) der Patienten festgestellt. Die größten Effekte traten zwischen der 10. und 24. Woche der Therapie auf.

### Männer mit genitalem LS

Kyriakou et al. kamen durch eine retrospektive Analyse zu dem Schluss, dass Clobetasolpropionat‐0,05 %‐Creme bei der Behandlung von genitalem LS bei Männern wirksam ist. Bei der anschließenden Erhaltungstherapie mit Methylprednisolonaceponat‐0,1 %‐Creme oder Tacrolimus‐0,1 %‐Salbe zeigten sich keine Unterschiede bezogen auf die Rückfallraten zwischen den beiden Präparaten.^82^

### Kinder mit genitalem LS

Es gibt nur wenige Fallserien, die über die Behandlung eines LS mit topischem Tacrolimus bei Kindern berichten. In kleineren Studien und Fallberichten zeigte sich unter Anwendung von 0,03  %^83^, ^84^ oder 0,1  %^85^ Tacrolimus‐Salbe eine klinische Besserung bis hin zur vollständigen Remission bei präpubertären Mädchen und Jungen. Eine Erhaltungstherapie mit reduzierter Applikationsfrequenz über mehrere Monate konnte Rückfälle reduzieren.^84^ Auch nach chirurgischen Eingriffen bei Jungen mit histologisch gesichertem LS erwies sich eine kurzzeitige adjuvante Tacrolimus‐Therapie als wirksam und gut verträglich.^86^

### Extragenitaler LS

Die alleinige Anwendung von topischem Tacrolimus bei extragenitalem LS erwies sich als erfolglos^73^, ^74^ oder topischen Glukokortikoiden unterlegen.^69^ Die Behandlung von extragenitalem LS mit Tacrolimus in Kombination mit UV‐Licht wurde erfolgreich eingesetzt.^74^, ^82^, ^87^

### Dosierung: Akut und Erhaltung

Es gibt keinen Konsens über das Behandlungsschema von genitalem LS mit topischen Calcineurininhibitoren. Der übliche Ansatz besteht darin, topische Calcineurininhibitoren zunächst zweimal täglich, möglicherweise gefolgt von einer einmal täglichen Anwendung, sobald sich die Läsionen zurückbilden, über insgesamt 3 bis 6 Monate kontinuierlich anzuwenden.^63^, ^65^

In einer retrospektiven Analyse von Anderson K et al. wurde berichtet, dass topische Calcineurininhibitoren bei Mädchen nach einer anfänglichen Behandlung mit topischem Clobetasolpropionat 0,05 % oder Mometasonfuroat 0,1 % ein‐ oder zweimal täglich über mehrere Wochen als Erhaltungstherapie eingesetzt werden können. Generell kann *off‐label* eine Deeskalation von topischen Glukokortikoiden auf topisches Tacrolimus bei Patienten mit LS empfohlen werden. Die topischen Glukokortikoide können währenddessen auf eine einmal tägliche Anwendung einmal pro Woche reduziert werden und ansonsten kann einmal täglich topisches Tacrolimus 0,1 % oder bei Kindern zunächst 0,03 % eingesetzt werden. Bei anhaltender Besserung der Läsionen kann die Anwendung von Clobetasolpropionat 0,05 % eingestellt und die Anwendung von Tacrolimus auf eine einmal tägliche Anwendung 1–2‐mal pro Woche reduziert werden.^88^

### Sicherheit

Topische Calcineurininhibitoren verursachen keine Hautatrophie, Hypopigmentierung, Striae, Teleangiektasien, *rebound flares* oder eine Suppression der Hypothalamus‐Hypophysen‐Nebennieren‐Achse. Die große Molekülgröße der topischen Calcineurininhibitoren minimiert ihre Absorption durch die Haut in den Blutkreislauf. Daher ist ihre Langzeitanwendung mit einer minimalen systemischen Absorption verbunden, wobei es in pharmakokinetischen Studien mit Erwachsenen und Kindern mit atopischem Ekzem keine Hinweise auf eine systemische Akkumulation gab.^75^ Die Blutkonzentrationen von Pimecrolimus wurden bei zehn Patientinnen überprüft und waren in allen Fällen nicht nachweisbar.^64^

Infektionen wie Herpes genitalis und vulvovaginale Candidiasis traten bei jeweils 2 % von 84 Patienten auf, die mit topischem Tacrolimus behandelt wurden. Während einer 18‐monatigen Nachbeobachtungszeit wurde keine Malignität beobachtet.^68^ In einem Fallbericht wurde die immunsuppressive Wirkung von topischem Tacrolimus 0,1 % bei einem zehnjährigen Mädchen mit LS in Zusammenhang mit einer bakteriellen Vaginose gebracht.^89^

Die theoretischen Sicherheitsbedenken (die in einer Tierstudie beobachtet wurden), dass topische Calcineurininhibitoren das Risiko von Lymphomen und anderen malignen Hauterkrankungen erhöhen könnten, werden in Fall‐Kontroll‐Studien, Meta‐Analysen und Post‐Marketing‐Registern nicht bestätigt.^76^, ^77^

### TOPISCHE THERAPIE MIT RETINOIDEN

**Table:** 

Bei *Frauen* mit genitalem LS *kann* eine Therapie mit topischen Retinoiden (off‐label) *erwogen werden*.	⇔	100 % Konsens evidenz‐ und konsensbasiert, siehe Evidenzbericht
Bei *Mädchen* mit genitalem LS *kann* eine Therapie mit topischen Retinoiden (off‐label) *erwogen werden*.	⇔	
Bei *Männern* mit genitalem LS *kann* eine Therapie mit topischen Retinoiden (off‐label) *erwogen werden*.	⇔	
Bei *Jungen* mit genitalem LS *kann* eine Therapie mit topischen Retinoiden (off‐label) *erwogen werden*.	⇔	
Bei Patienten mit *extragenitalem LS kann* eine Therapie mit topischen Retinoiden (off‐label) *erwogen werden*.	⇔	

### Einführung

Retinoide induzieren sowohl in der Dermis als auch in der Epidermis Veränderungen. Viele ihrer Effekte werden durch ihre Interaktion mit nukleären Retinoidsäurerezeptoren (RAR) und Retinoid‐X‐Rezeptoren (RXR) vermittelt.^90^ Ein Ungleichgewicht in der Expression nukleärer RAR (RAR‐α und RAR‐γ) wurde bei der Pathogenese des LS der Vulva postuliert.^91^

### Wirksamkeit und Wirkmechanismus

Es gibt nur wenige Fallserien, die über topische Retinoide bei der Behandlung des LS berichten.

Die Evidenzlage zur Wirksamkeit topischer Retinoide bei der Behandlung des vulvären LS ist begrenzt und basiert überwiegend auf kleinen Fallserien und unkontrollierten Studien. In einer offenen Studie mit 22 Patientinnen zeigte eine einjährige Anwendung von 0,025  % Tretinoin eine Besserung subjektiver Symptome wie Juckreiz, Brennen und Dyspareunie sowie objektiver Befunde (zum Beispiel Hyperkeratose, Sklerose).^92^ Auch 13‐cis‐Retinsäure (0,5  %) und 0,025%iges topisches Tretinoin führte in einer weiteren Fallserie zu einer vollständigen oder teilweisen Rückbildung der Symptome.^93^, ^94^ Eine retrospektive Kohortenstudie konnte keinen zusätzlichen Nutzen der Kombination von Tretinoin mit Mometasonfuroat gegenüber Mometasonfuroat allein nachweisen.^95^

### Sicherheit

Nebenwirkungen, hauptsächlich leichte Erytheme und Brennen, werden bei etwa 35 % der Patienten berichtet.^94^ Selten brachen Patienten die Behandlung aufgrund von Nebenwirkungen ab.

### Besondere Aspekte

Topische Retinoide sind in der Schwangerschaft und bei geplanter Schwangerschaft kontraindiziert. Wenn topische Retinoide im gebärfähigen Alter angewandt werden, müssen sie bei eingetretener Schwangerschaft abgesetzt werden, eine Ultraschalluntersuchung, die gezielt auf Fehlbildungen untersucht, sollte angeboten werden.

### TOPISCHE THERAPIE MIT HORMONEN

**Table:** 

Bei *Frauen soll* topisches *Testosteron* oder *Dihydrotestosteron* zur Behandlung eines genitalen LS *nicht* eingesetzt werden.	**↓↓**	100 % Konsens evidenz‐ und konsensbasiert, siehe Evidenzbericht
Bei *Frauen soll* topisches *Progesteron* zur Behandlung eines genitalen LS *nicht* eingesetzt werden.	**↓↓**	
Bei *Frauen soll* topisches *Östrogen* zur Behandlung eines genitalen LS *nicht* eingesetzt werden.*	**↓↓**	
Bei *Mädchen sollen* topische Hormon‐Präparate zur Behandlung eines genitalen LS *nicht* eingesetzt werden.	**↓↓**	
Bei *Männern sollen* topische Hormon‐Präparate zur Behandlung eines genitalen LS *nicht* eingesetzt werden.	**↓↓**	
Bei *Jungen sollen* topische Hormon‐Präparate zur Behandlung eines genitalen LS *nicht* eingesetzt werden.	**↓↓**	
Bei Patienten *sollen* topische Hormon‐Präparate zur Behandlung eines *extragenitalen LS nicht* eingesetzt werden.	**↓↓**	
*Bei Frauen, die unter einem zusätzlichen Urogenitalsyndrom leiden, kann eine topische vaginale Östrogen‐Behandlung jedoch hilfreich sein.		

### PLATELET RICH PLASMA

**Table:** 

Bei *Frauen* mit genitalem LS *kann* eine Therapie mit *platelet rich plasma erwogen werden*.	⇔	> 75 % Konsens evidenz‐ und konsensbasiert, siehe Evidenzbericht
Bei *Mädchen soll* eine Therapie des genitalen LS mit *platelet rich plasma nicht erfolgen*.	**↓↓**	
Bei *Männern* mit genitalem LS *kann* eine Therapie mit *platelet rich plasma erwogen werden*.	⇔	
Bei *Jungen soll* eine Therapie des genitalen LS mit *platelet rich plasma nicht erfolgen*.	**↓↓**	
Bei *Patienten mit extragenitalem LS soll* eine Therapie mit *platelet rich plasma nicht erfolgen*.	**↓↓**	

### UV‐THERAPIE

**Table:** 

Bei *Frauen* mit genitalem LS *kann* eine UV‐Therapie *erwogen werden*.	⇔	100 % Konsens evidenz‐ und konsensbasiert, siehe Evidenzbericht
Bei *Mädchen soll* eine UV‐Therapie zur Behandlung eines genitalen LS *nicht* eingesetzt werden	**↓↓**	
Bei *Männern* mit genitalem LS *kann* eine UV‐Therapie *erwogen werden*.	⇔	
Bei *Jungen soll* eine UV‐Therapie zur Behandlung eines genitalen LS *nicht* eingesetzt werden.	**↓↓**	
Bei Patienten mit *extragenitalem LS soll* eine UV‐Therapie eingesetzt werden.	**↑↑**	

### Wirksamkeit und Wirkmechanismus

Die Phototherapie, insbesondere mit ultraviolettem (UV)A‐Licht, ist eine wirksame und gut etablierte Behandlungsoption für sklerosierende Hauterkrankungen wie Morphea, Hautbeteiligung bei systemischer Sklerose oder sklerodermiforme Graft‐versus‐Host‐Erkrankung. Für verschiedene sklerosierende Erkrankungen der Haut wurden zahlreiche Studien (einschließlich prospektiver kontrollierter Studien) durchgeführt.^96^, ^97^, ^98^, ^99^, ^100^ Im Gegensatz dazu gibt es nur wenige Daten über die Wirksamkeit der UV‐Therapie bei genitalem LS. Die meisten Daten basieren auf kleinen Fallserien oder Einzelfallberichten.^101^, ^102^, ^103^

### Dosierung

UVA1‐Phototherapie sollte mit niedriger (20 J/cm^2^) oder mittlerer Dosis (50 J/cm^2^) für insgesamt maximal 40 Anwendungen pro Zyklus erfolgen.

### Sicherheit

Im Allgemeinen ist UVA1 gut verträglich, zu den Nebenwirkungen können ein frühes Erythem direkt nach der Bestrahlung, eine Bräunung der bestrahlten Haut sowie Juckreiz oder Brennen kurz nach der Behandlung gehören.

### Überwachung

Wenn möglich, sollte eine klinische Untersuchung während der UVA1‐Behandlung wöchentlich oder alle 14 Tage durchgeführt werden.

### UV‐Therapie bei extragenitalem LS

Es gibt ebenfalls nur wenige Studien zur Sicherheit und Wirksamkeit von UV‐Licht bei extragenitalem LS.^104^, ^105^, ^106^ Die Wirksamkeit der UVA1‐Phototherapie bei extragenitalem LS wurde erstmals 2001 von Kreuter et al. berichtet.^105^ Die Autoren berichteten über eine Verbesserung der LS bedingten Läsionen bei zwei Patientinnen nach 40 Sitzungen UVA1‐Bestrahlung (vier Sitzungen pro Woche über 10 Wochen, insgesamt 40 Behandlungen, 20 J/cm^2^ niedrig dosiertes UVA1 pro Sitzung, 800 J/cm^2^ kumulative Dosis). Ein Jahr später konnten Kreuter et al. die Wirksamkeit von UVA1 bei extragenitalem LS bei zehn Patienten präsentieren, die alle mit dem etablierten Standard‐Bestrahlungsprotokoll behandelt wurden.^106^, ^107^

Was die Bade‐PUVA‐Therapie bei extragenitalem LS betrifft, so zeigte ein Fallbericht mit einer verwendeten kumulativen Dosis von 31,7 J/cm^2^ und Einzeldosen zwischen 0,3–2,3 J/cm^2^ ein vielversprechendes Ergebnis.^108^

Die Schmalband (NB)‐UVB‐Phototherapie allein oder in Kombination mit Salzwasser (Balneophototherapie) ist eine etablierte Therapie bei Psoriasis. Ähnlich wie bei UVA1 gibt es nur wenige Fallberichte über NB‐UVB bei extragenitalem LS.^104^, ^109^ Eine große randomisierte kontrollierte Studie bei Patienten mit Morphea hat jedoch gezeigt, dass die NB‐UVB einen positiven Einfluss auf sklerotische Hautveränderungen hatte, aber der UVA1‐Therapie signifikant unterlegen war.^110^ Aufgrund dieser Ergebnisse könnte die NB‐UVB‐Therapie als alternative Behandlung für extragenitalen LS in Zentren in Betracht gezogen werden, in denen UVA1 nicht verfügbar ist. Der Einsatz von NB‐UVB beim vulvären LS wurde unseres Wissens nach bisher nur in Einzelfallberichten als wirksam beschrieben.^111^

Vorsicht ist geboten bei Patienten mit zusätzlichen Kollagenosen, wie zum Beispiel Lupus erythematosus, die eine Kontraindikation für eine UV‐Behandlung darstellen können.^112^

### PHOTODYNAMISCHE THERAPIE

**Table:** 

Bei *Frauen* mit genitalem LS *kann* eine photodynamische Therapie *erwogen werden*.	⇔	100 % Konsens evidenz‐ und konsensbasiert, siehe Evidenzbericht
Bei *Mädchen soll* eine photodynamische Therapie zur Behandlung eines genitalen LS *nicht* eingesetzt werden.	**↓↓**	
Bei *Männern* mit genitalem LS *kann* eine photodynamische Therapie *erwogen werden*.	⇔	
Bei *Jungen soll* eine photodynamische Therapie zur Behandlung eines genitalen LS *nicht* eingesetzt werden.	**↓↓**	
Bei Patienten mit *extragenitalem LS kann* eine photodynamische Therapie *erwogen werden*.	⇔	

### LASER‐THERAPIE

**Table:** 

Bei *Frauen* mit genitalem LS *kann* eine Therapie mit *fraktionierten ablativen CO_2_‐Laser erwogen werden*.	⇔	100 % Konsens evidenz‐ und konsensbasiert, siehe Evidenzbericht
Bei *Frauen* mit genitalem LS *kann* zur Behandlung der Gewebeverhärtung eine Therapie mit *nicht‐ablativen Nd:YAG‐Laser erwogen werden*.	⇔	
Bei *Männern* mit genitalem LS *kann* eine Therapie mit *fraktionierten ablativen CO_2_‐Laser erwogen werden*.	⇔	
Bei *Männern* mit genitalem LS *kann* zur Behandlung der Gewebeverhärtung eine Therapie mit *nicht‐ablativen Nd:YAG‐Laser erwogen werden*.	⇔	
Bei Patienten mit *genitalem LS kann* eine *kombinierte Laser Therapie* (zum Beispiel ablative und nicht‐ablative Laser) *erwogen werden*.	⇔	
Bei *Kindern soll* eine Lasertherapie zur Behandlung eines LS *nicht* eingesetzt werden.	**↓↓**	

### KRYOTHERAPIE

**Table:** 

Bei *Frauen sollte* eine Kryotherapie zur Behandlung eines genita LS *nicht* eingesetzt werden.	**↓**	100 % Konsens evidenz‐ und konsensbasiert, siehe Evidenzbericht
Bei *Mädchen soll* eine Kryotherapie zur Behandlung eines genitalen LS *nicht* eingesetzt werden.	**↓↓**	
Bei *Männern soll* eine Kryotherapie zur Behandlung eines genitalen LS *nicht* eingesetzt werden.	**↓↓**	
Bei *Jungen soll* eine Kryotherapie zur Behandlung eines genitalen LS *nicht* eingesetzt werden.	**↓↓**	
Bei Patienten mit *extragenitalem LS soll* eine Kryotherapie zur Behandlung *nicht* eingesetzt werden.	**↓↓**	

### SYSTEMISCHE THERAPIE

**Table:** 

Wenn bei *Frauen* mit genitalem LS eine systemische Therapie erforderlich ist, *sollte* diese, unter Berücksichtigung der Teratogenität, mit *Acitretin* (off‐label) erfolgen.	**↑**	100 % Konsens evidenz‐ und konsensbasiert, siehe Evidenzbericht
Wenn bei *Männern* mit genitalem LS eine systemische Therapie erforderlich ist, *sollte* diese mit *Acitretin* (off‐label) erfolgen.	**↑**	> 75 % Konsens evidenz‐ und konsensbasiert, siehe Evidenzbericht
Wenn bei *erwachsenen Patienten* mit *genitalem und/oder extragenitalem LS* eine systemische Therapie erforderlich ist, *sollte* diese, unter Berücksichtigung der Teratogenität, mit Methotrexat (off‐label) erfolgen.	**↑**	100 % Konsens evidenz‐ und konsensbasiert, siehe Evidenzbericht

### Einführung

Eine systemische Behandlung eines LS wurde bereits mit verschiedenen Wirkstoffen versucht. Die Evidenzlage ist insgesamt sehr gering und die Medikamente wurden meist nicht bei allen Formen von LS eingesetzt.

#### Systemische Retinoide

#### Wirksamkeit und Wirkmechanismus

Es gibt mehrere Fallserien sowie zwei randomisierte kontrollierte Studien, die über die Behandlung von LS mit oralen Retinoiden berichten. In einer unkontrollierten Studie beobachteten Mørk et al. bei sechs von acht Patientinnen mit behandlungsresistentem LS der Vulva unter oraler Gabe von Etretinat (1 mg/kg/Tag) nach 14–18 Wochen eine Verbesserung der klinischen Symptome (Outcome‐Erhebung durch Patientinnen und Ärzte).^113^ Romppanen et al. behandelten 19 Frauen mit LS der Vulva 3 Monate lang mit oralem Etretinat (Anfangsdosis 0,54 mg/kg/Tag, Erhaltungsdosis 0,26 mg/kg/Tag). In der Gruppe mit schwerer Vulvadystrophie wurde in fast allen Fällen eine Verringerung des Schweregrads erreicht.^114^ Darüber hinaus wurden zwei kleine doppelblinde placebokontrollierte Studien zur Behandlung von genitalem LS mit Acitretin veröffentlicht.^115^, ^116^ In einer multizentrischen, doppelblinden Studie behandelten Bousema et al. Patientinnen (78 wurden in die Studie aufgenommen, 46 wurden in die Wirksamkeitsanalyse eingeschlossen) mit LS der Vulva mit 20 bis 30 mg Acitretin oder Placebo über insgesamt 16 Wochen. Die Symptome und klinischen Zeichen verbesserten sich sowohl in der Behandlungsgruppe als auch in der Placebogruppe. In der Acitretin‐Gruppe zeigte sich jedoch eine geringere Intensität aller Symptome sowie geringere klinische Zeichen. Statistisch signifikante Unterschiede traten bei Pruritus, Atrophie und Hyperkeratose auf.^115^ In einer weiteren randomisierten kontrollierten Studie von Ioannides et al. wurden 52 männliche Patienten mit schwerem, langjährigem LS im Verhältnis 2:1 randomisiert und erhielten 20 Wochen lang Acitretin (35 mg) oder Placebo. Der mittlere klinische Gesamtscore der Acitretin‐Gruppe war signifikant niedriger als der der Kontrollgruppe, was auch mit einer signifikanten Verbesserung des mittleren DLQI einherging.^116^ Aus diesen Ergebnissen schlossen die Autoren, dass Acitretin bei langjährigem LS bei Männern wirksam sei.^115^

#### Sicherheit

Retinoide werden in der Regel gut vertragen, jedoch tritt häufig eine Haut‐ und Schleimhauttrockenheit als Nebenwirkung auf. Cholesterin‐, Triglycerid‐ und Transaminasen können während der Therapie ansteigen und sollten vor und während der Therapie regelmäßig kontrolliert werden. Systemische Retinoide sind hochgradig teratogen, daher müssen alle Frauen im gebärfähigen Alter eine sichere Empfängnisverhütung anwenden (während der Therapie und, je nach Präparat, bis zu drei Jahre nach Beendigung der Therapie).

### Methotrexat

#### Wirksamkeit und Wirkmechanismus

Methotrexat (MTX), ein strukturelles Analogon der Folsäure, wirkt durch Hemmung des Folsäurestoffwechsels. Es wird bei der Behandlung von Neoplasien und entzündlichen Erkrankungen eingesetzt. In einer retrospektiven Fallserie wurden 28 Patienten mit LS (24/28 mit extragenitaler Beteiligung) beschrieben, die mit MTX 2,5 bis 17,5 mg wöchentlich behandelt wurden. In 21/28 Fällen kam es zu einer anfänglichen Verbesserung des LS und in 15 Fällen zu einer anhaltenden Verbesserung. Die meisten Patienten wurden in Kombination mit topischen Glukokortikoiden oder Tacrolimus behandelt.^117^ In einer anderen Studie wurden sieben Patienten mit generalisiertem LS (5 Patienten mit genitaler und extragenitaler Beteiligung; 2 ohne genitale Beteiligung) mit hochdosiertem intravenösem Methylprednisolon behandelt, das als Einzeldosis von 1000 mg an drei aufeinanderfolgenden Tagen monatlich verabreicht wurde, plus MTX 15 mg/Woche (oral) für mindestens 6 Monate (maximal 10 Monate). Alle Patienten waren zuvor erfolglos mit topischen Glukokortikoiden und UV‐Phototherapie behandelt worden. Der extragenitale LS verbesserte sich bei Patienten in der Regel nach dreimonatiger Behandlung; eine 100%ige Heilung wurde nicht erreicht. Über die Wirkung auf genitale Läsionen wurde nicht berichtet. Die beobachteten unerwünschten Wirkungen (Übelkeit bei 3 Patienten, Kopfschmerzen bei 3 Patienten und ein 2‐facher Anstieg der Leberenzymwerte bei einem Patient) waren mäßig und verschwanden nach Abschluss der Behandlung.^118^ Eine Patientin mit generalisiertem LS, der die extragenitale Haut und den Anogenitalbereich betraf, wurde 8 Monate lang erfolgreich mit MTX 10 mg/Woche behandelt; eine Besserung wurde nach 3 Wochen und ein ausgezeichnetes Ansprechen nach 5 Monaten festgestellt.^119^

#### Dosierung

Methotrexat in einer Dosierung von 10 bis 15 mg/Woche (subkutan oder oral) über einen Zeitraum von 6 Monaten, möglicherweise in Kombination mit systemischen Glukokortikoiden, kann behandlungsresistente, generalisierte Formen des LS verbessern.

#### Sicherheit

Die häufigsten Nebenwirkungen sind gastrointestinale Beschwerden, Kopfschmerzen, Müdigkeit, Stimmungsschwankungen^120^ und erhöhte Leberenzyme. Panzytopenien treten hauptsächlich bei Überdosierung auf. Über eine MTX induzierte Alveolitis wurde selten berichtet. Vor Beginn einer MTX‐Therapie müssen chronische Infektionen (wie Hepatitis B/C, HIV, Tuberkulose) ausgeschlossen werden. Impfungen sollten entsprechend den örtlichen Empfehlungen verabreicht werden. Lebendimpfungen sind kontraindiziert. Ein Differenzialblutbild sowie ein Nieren‐ und Leberprofil sind vor und regelmäßig während der Therapie erforderlich. Nationale Leitlinien sollten zu Rate gezogen werden.

## CHIRURGISCHE INTERVENTIONEN

**Table:** 

Bei *Frauen* mit genitalem LS, die trotz leitliniengerechter Behandlung mit topischen Glukokortikoiden eine persistierende Stenose des Introitus vaginae haben, welche mechanische Probleme bei der Miktion oder beim Geschlechtsverkehr verursacht, *sollte* eine *Adhäsiolyse / Synechiolyse / Perineoplastik oder anatomische Vulvaplastik* durchgeführt werden.	**↑**	100 % Konsens Konsensbasiert*
Bei *Mädchen sollten* chirurgische Interventionen zur Behandlung eines genitalen LS *nicht* durchgeführt werden, können im Einzelfall jedoch zur Verbesserung funktioneller Störungen indiziert sein.	**↓**	100 % Konsens Konsensbasiert*
Bei *Männern und Jungen* mit LS bedingter Phimose, die nicht auf eine leitliniengerechte Behandlung, zum Beispiel mit topischen Glukokortikoiden, angesprochen hat, *soll* eine *Zirkumzision*, vorzugsweise mit Entfernung der gesamten Vorhaut, erfolgen.	**↑↑**	> 75 % Konsens Konsensbasiert*
Bei *Männern und Jungen* mit LS bedingter isolierter Verkürzung des Frenulums, die nicht auf eine leitliniengerechte Behandlung, zum Beispiel mit topischen Glukokortikoiden, angesprochen hat, *sollte* eine *Frenuloplastik* mit lokaler Triamcinoloninjektion oder Nachbehandlung in Form lokaler Glukokortikoide erfolgen.	**↑**	100 % Konsens Konsensbasiert*
Bei *Männern und männlichen Jugendlichen* mit LS bedingten Harnröhrenstrikturen, die mechanische Probleme bei der Miktion oder beim Geschlechtsverkehr verursachen, *soll* eine *Urethroplastik* mit Mundschleimhauttransplantat durchgeführt werden.	**↑↑**	100 % Konsens Konsensbasiert*
Bei *Männern und Jungen* mit LS‐bedingter Meatusstenose, die nicht auf eine leitliniengerechte Behandlung mit topischen Glukokortikoiden angesprochen hat, *sollte* eine Meatusplastik erfolgen.	**↑**	100 % Konsens Konsensbasiert*
Bei *Männern und Jungen* mit LS‐bedingter Urethra‐ oder Meatusstenose *sollte* eine Bougierung außer zu palliativen Zwecken *nicht* erfolgen.	**↓**	100 % Konsens Konsensbasiert*
*Aufgrund der geringen Evidenzlage entschied sich das Leitliniengremium hier rein Konsens‐basierte Empfehlungen trotz systematischer Evidenzaufbereitung (siehe Evidenzbericht) auszusprechen.		
Vor einer chirurgischen Intervention *sollen* Frauen … − über die notwendige topische Behandlung (in der Regel mit Glukokortikoid‐Salben (meist Clobetasolpropionat 0,05 %) präoperativ und postoperativ informiert werden und dieser zustimmen. − wenn möglich eine interdisziplinäre Beratung, einschließlich durch spezialisierte Beckenbodenphysiotherapeuten und Sexualtherapeuten, erhalten. Bei chirurgischen Interventionen bei Männern … − *soll* die entfernte Vorhaut histopathologisch untersucht werden, um den LS zu verifizieren und präkanzeröse Läsionen auszuschließen. − *soll* prä‐ und postoperativ eine Behandlung mit topischen Glukokortikoid‐Salben (meist Clobetasolpropionat 0,05 %) erfolgen (zum Beispiel 4 Wochen vor und 4‐12 Wochen nach dem Eingriff). − *sollte* die Vorhaut bei einer Zirkumzision vollständig entfernt werden.	**↑↑**	100 % Konsens konsensbasiert
*sollte* die Vorhaut bei einer Zirkumzision vollständig entfernt werden.	**↑**	

## BESONDERHEITEN DES EXTRAGENITALEN LS

Im Vergleich zum genitalen LS ist die extragenitale Manifestation des LS in unseren Breiten viel seltener, sie betrifft etwa 10 %–20 % der Patienten mit LS.^9^ Sie tritt vorwiegend bei Frauen auf, wobei das Verhältnis zwischen Frauen und Männern mit 7:2 beschrieben wird.^121^ Ein extragenitaler LS ohne gleichzeitige genitale Manifestation ist bei uns sehr selten. Der extragenitale LS und Morphea weisen klinische Ähnlichkeiten auf, und es wird über eine intraindividuelle Koexistenz beider Erkrankungen berichtet.^122^ Klinisch zeigt sich der extragenitale LS als porzellanartige polygonale Papeln oder Plaques (Abbildung 5). Für den extragenitalen LS sind mehrere morphologische Varianten beschrieben, darunter der bullöse, ulzerative, ringförmige, desquamative, teleangiektatische, angiokeratomatöse, verruköse und vitiligoide LS.^121^ Beim bullösen extragenitalen LS könnte die Blasenbildung durch zwei Mechanismen erklärt werden. Zum einen ist die Stabilität der Basalmembranzone durch die Interface‐Dermatitis und Apoptose der Basalzellschicht gestört. Zum anderen führt das Ödem der papillären Dermis zu einem Auseinanderweichen der Kollagenfasern und zur Abflachung der Reteleisten.^123^ Die meisten Läsionen sind asymptomatisch oder sind von leichtem Juckreiz begleitet. Bei ausgedehntem extragenitalen LS können jedoch Hautatrophie und Sklerose erhebliche Beschwerden verursachen. Das Köbner Phänomen ist beim LS beschrieben. So wurde kürzlich über das Auftreten eines extragenitalen LS in einer Tätowierung berichtet.^124^ Der extragenitale LS kann den gesamten Körper betreffen, die besonders prädisponierten Lokalisationen sind aber vor allem der Rumpf (Submammär‐Region, Bauch, Gesäß, Schultern, Handgelenke und Brust) sowie die proximalen Extremitäten.^124^, ^125^ In sehr seltenen Fällen wurde auch eine Beteiligung der Mundschleimhaut (überwiegend der Lippen und bukkal) beschrieben.^126^, ^127^ Diese Fälle sind jedoch kritisch zu hinterfragen, da aufgrund der klinischen und histopathologischen Ähnlichkeiten ein oraler Lichen planus als Manifestationen eines LS in der Mundhöhle fehlgedeutet werden kann und beide Erkrankungen parallel auftreten können.

Ein kürzlich erschienener Bericht aus dem Irak beschreibt die vorwiegend extragenitale Manifestation des LS. Dies wirft die Frage nach einem unterschiedlichen genetischen Hintergrund oder unterschiedlichen Triggerfaktoren auf.^27^

## NACHSORGE

**Table:** 

Patienten mit LS *sollen* eine regemäßige Nachsorge erhalten; anfangs zum Beispiel alle 3–6 Monate und nach Stabilisierung der Erkrankung zum Beispiel alle 12 Monate.	**↑↑**	100 % Konsens konsensbasiert

**Table:** 

Kinder mit LS *sollen* durch spezialisierte Ärzte, die Erfahrung mit LS bei Kindern haben, nachgesorgt werden.	**↑↑**	100 % Konsens konsensbasiert

**Table:** 

Erwachsene Patienten mit LS, die nicht auf eine topische Therapie mit Glukokortikoiden der Klasse III oder IV ansprechen oder die präkanzeröse Läsionen der Vulva oder des Penis aufweisen, *sollen* durch spezialisierte Ärzte, wie zum Beispiel Dermatologen, Gynäkologen oder Urologen nachgesorgt werden.	**↑↑**	100 % Konsens konsensbasiert

**Table:** 

Patienten mit LS, bei denen Miktionsbeschwerden auftreten, *sollen* zu entsprechenden Experten überwiesen werden.	**↑↑**	100 % Konsens konsensbasiert

**Table:** 

Die Nachsorge von Patienten mit LS *soll* folgendes beinhalten: − Überwachung der Wirksamkeit der Behandlung (einschließlich Linderung/Kontrolle der Symptome, Normalisierung der Hautfarbe und ‐beschaffenheit), − Erfragen von Problemen bei der Miktion, der Defäkation und der Sexualfunktion, − engmaschige Überwachung der Entwicklung von präkanzerösen oder kanzerösen Läsionen, − Sicherstellung/Gewährleistung der Therapietreue/Compliance.	**↑↑**	100 % Konsens konsensbasiert

## INTERDISZIPLINÄRES MANAGEMENT

**Table:** 

Überweisungen zu weiteren Fachkräften *sollen* zum Beispiel in folgenden Situationen erfolgen: − wenn nach adäquater Behandlung keine ausreichende Verbesserung der klinischen Zeichen und/oder der Symptome festgestellt wird; − wenn Komplikationen auftreten, die spezialisierte Ansätze erfordern, etwa funktionelle Beeinträchtigungen, die eine chirurgische Behandlung erfordern, oder chronische Schmerzsyndrome, die von Schmerztherapeuten betreut werden müssen; − wenn psychologische Unterstützung benötigt wird; − wenn sexualtherapeutische Unterstützung benötigt wird und − wenn die Transition von Jugendlichen in die Erwachsenenmedizin indiziert ist.	**↑↑**	100 % Konsens konsensbasiert

## DANKSAGUNG

Open access Veröffentlichung ermöglicht und organisiert durch Projekt DEAL.

## INTERESSENKONFLIKT

Eine vollständige Übersicht der erklärten Interessenkonflikte ist im Leitlinienreport verfügbar unter https://register.awmf.org/de/leitlinien/detail/013‐105
